# Exploring dual career quality implementation at European higher education institutions: Insights from university experts

**DOI:** 10.1371/journal.pone.0277485

**Published:** 2022-11-30

**Authors:** Pascal Izzicupo, Angela Di Baldassarre, Barbara Ghinassi, Ilvis Abelkalns, Ugis Bisenieks, Antonio Sánchez-Pato, Francisco José Cánovas-Alvarez, António J. Figueiredo, Juan Alfonso García-Roca, Alejandro Leiva-Arcas, Lourdes Meroño, Anda Paegle, Liliana-Elisabeta Radu, Cristian-Mihail Rus, Oana-Mihaela Rusu, Hugo Sarmento, Janis Stonis, Raquel Vaquero-Cristóbal, Vasco Vaz, Mojca Doupona, Laura Capranica, on behalf of the More Than Gold Consortium

**Affiliations:** 1 Department of Medicine and Aging Sciences, University “G. D’Annunzio” of Chieti-Pescara, Chieti CH, Italy; 2 University of Latvia, Riga, Latvia; 3 European Athlete as Student Network, Ghaxaq, Malta; 4 Olympic Studies Center, Faculty of Sport, Universidad Católica San Antonio, Murcia, Spain; 5 Research Unit for Sport and Physical Activity (CIDAF), Faculty of Sport Sciences and Physical Education, University of Coimbra, Coimbra, Portugal; 6 Faculty of Physical Education and Sport, University "Alexandru Ioan Cuza" of Iași, Iași, Romania; 7 Faculty of Sport, Injury prevention in Sport Research Group, UCAM, Murcia, Spain; 8 Faculty of Sports, Department of Sport Sociology and History, University of Ljubljana, Ljubljana, Slovenia; 9 Department of Movement, Human and Health Sciences, University of Rome Foro Italico, Rome, Italy; Polytechnic Institute of Coimbra: Instituto Politecnico de Coimbra, PORTUGAL

## Abstract

**Introduction:**

This paper examines the convergence of the opinions of European higher education institution (HEI) experts on possible implementation of dual career policies and provision areas at university level.

**Methods:**

An online 32-item questionnaire encompassing 26 dual career aspects collected the opinion of European HEI experts as the last phase of a Delphi method preceded by i) focus groups with student-athletes who aimed to identify needs for dual careers and ii) a workshop with 21 dual career experts to generate the statements to be included in the survey. Seventy-one HEI experts from 12 EU member states participated in the survey, rating each dual career aspect identified in previous phases.

**Results:**

The relative position of each aspect has been plotted based on relevance (x-axis) and feasibility (y-axis). The Quadrant IV of the resulting scatterplots identified the following nine aspects rated as highly relevant and highly feasible for implementation: tutorship/mentorship, psychological support, programmes based on integration of academic departments and sports services, and adaptable programmes to the needs of each student-athletes (assistance/tutorship area), individual study plans and distance learning (curricula requirements area), publicity for student-athletes and initiatives for increasing the awareness of student-athletes and knowledge of dual career issues (social support area), and access to educational facilities (logistic support area).

**Discussion and conclusions:**

The HEI experts’ views represent a coherent and useful starting point to develop a deep understanding of the considered 26 aspects founded on a phenomenological lifeworld-led approach and emphasizes the need for a minimum standard for dual career policies and provisions.

## Introduction

Considering that the Member States have full competences in the education and sports areas, the European Union adopts a non-directive policy to promote and support coordinated intergovernmental cooperation for a coherent approach towards the development of these areas [1, 2]. In the last decades, the Bologna Process and the EuRopean community Action Scheme for the Mobility of University Students (ERASMUS) generated structured cooperation among European higher education institutions (HEIs), including student, teacher, and staff mobility, uniformity of accreditation and duration of degrees, organization of academic years, curricula content and teaching methods, credit transfer and accumulation system (ECTS), and service provision [3–6]. However, differences between the Member States exist at the HEIs and the sports levels in relation to policies and organizations. In 2009, the amended Treaty of the European Union recognized the ‘specificity of sport’. Based on the specificity principle, sports bodies independently set organizational structures and rules to organize clubs, competitions, and championships at local, national, regional, and international levels [7].

Talented and elite athletes have the right to pursue their sport and education careers (i.e., dual career). However, in considering the various arrangements at educational and sports levels, student-athletes might need support from multiple stakeholders when academic and sport commitments conflict [7–9]. At present, important disparities exist in dual career support between the Member States due to national cultural and organizational aspects. According to Aquilina and Henry [10], the Member States approach the dual carrier in different ways that can be categorized as (1) State-centric regulation, in which government legislation or statutory regulations place responsibilities on HEIs to provide flexible academic paths; (2) State as a sponsor/facilitator, in which states promote formal agreements to meet athletes’ needs at the educational level; (3) National Sporting Federation/Institute as an intermediary, in which national governing or sports bodies negotiate flexible academic paths with educational institutions; and (4) Laisser-faire/No Formal Structures, in which individually negotiated agreements are arranged, when possible. In the latter case, sports and education are considered distinct and separate, often impairing the holistic development of student-athletes and interfering with or disrupting favourable transfer of knowledge and experiences between careers [11]. Furthermore, the lack of a clear and consistent definition of “student-athlete” determines different eligibility criteria for dual career programmes and services between Member States [7, 8]. To overcome inter- and intra-country differences, dual career of athletes is one of the priorities of the European strategy, and Member States are envisaged to promote and carry out actions in support of the holistic development of talented and elite student-athletes according to the EU Guidelines on Dual Careers of Athletes published in 2012 [9, 12–14]. Specifically, HEIs are urged to define a dual career vision and consequent strategies and policies to support athletes encompassing: flexible educational programmes and examination schedules; blended and distance learning; tutorship and counselling; living, educational, and sports facilities at a close distance; recognition of ECTS for skills acquired in and through sports participation; and plenty visibility on media and social media [7, 9, 15]. After ten years of the publication of EU Guidelines, dual career services still lack minimum standards, and student-athletes suffer a limited implementation of dual career measures [7, 16].

For sharing best practices and for envisaging innovative solutions to bridge the gap between policies and provisions for athletes combining their sports and academic careers, in the last decade the European Commission allocated funds for ERASMUS+Sport Collaborative Partnerships and the lifelong learning education programme to foster the cooperation between dual career sport and education stakeholders [17, 18]. Furthermore, an increasing number of studies focused on the development of dual career, also at HEI level [19, 20]. For example, Morris et al. [21] provided a taxonomy for dual career development environments in European Countries, identifying eight different environments ranging from sport-friendly to professional and national HEIs. On the other hand, Storm et al. [22] identified ten essential features of European dual career development environments which can help researchers and practitioners to optimize those environments for student-athletes. Furthermore, in a recent analysis by Hong et al. [23] aiming to identify support/services for junior athletes, financial support emerged as the most prevalent type of support. In contrast, other types such as tutoring, academic flexibility, support from sports psychologists, nutritionists, strength and conditioning coaches, and dual career management were furnished in different combinations. Whilst it is important to know the services offered for dual career development in the European Union, a large body of research focused on both users (i.e., student-athletes) and providers (i.e., workers in higher education institutions). For this purpose, Defruyt et al. [24] developed a questionnaire on competences for dual career support providers. Notwithstanding, due to the differences in both sports and educational legislation, as well as in the dual career approach across European Union, the actual match between the students’ demand for services and the supply by the HEIs, which involve various actors at different levels, needs to be further elucidated to identify the areas of intervention and the importance of the services to be offered and their feasibility.

To address the complex policy question of establishing effective mechanisms for the harmonization of dual career within the European HEIs, the European-funded Collaborative Partnership “More Than Gold” (MTG, 603346-EPP-1-2018-1-LV-SPO-SCP) aimed to examine strategies for the best conditions to promote dual career management at HEIs. Comparing experiences in different Member States and sharing local/national best practices could help envisaging transnational dual career policies [10, 25]. Therefore, the MTG team used an ethnographic research approach, which helps to describe a group or culture, strongly relying on personal experiences to make additional decisions about which approaches are appropriate for the situation at hand [26]. To develop specific guidelines for implementing dual career policies and provisions at the HEI level, a participatory approach and an active engagement of university athletes and academic experts was deemed crucial to gather a comprehensive understanding of key aspects, structures, and their interrelationships. Thus, a `Delphi’ multistage consensus method was used, which encompassed: i) national focus groups with university dual careers athletes to identify their needs; ii) a workshop with 21 European dual career experts to identify dual career services at HEI corresponding to the needs of the student-athletes; and iii) the administration of a survey to HEIs experts to verify the convergence of their opinions on the importance and feasibility of dual career services [27]. The first two phases of this iterative multistage process designed to transform opinion into group consensus gathered insights into the needs of university student-athletes through focus groups organized at five European universities under national dual career policies presenting State-centric regulations (e.g., University of Coimbra, Portugal; and UCAM Catholic University of Murcia, Spain), State as sponsor/facilitator (e.g., University of Latvia, Latvia) and laisser faire-no formal structure (e.g., University “G. D’Annunzio” of Chieti-Pescara, Italy; and University "Alexandru Ioan Cuza" of Iași, Romania). Thus, dual career aspects emerging from the first phase were discussed during a workshop with 21 dual career experts, who established trustworthiness and analyzed all meaning units, themes, and categories [28, 29]. Thus, inductive thematic analysis was deemed necessary for organizing and interpreting the recorded statements into content units [30, 31], resulting in six thematic areas (e.g., Financial Support, Logistic Support, Assistance/Tutorship, Curricula Requirements, Social Support, and Policies), and reached a consensus [32].

To help enhancing effective decision-making in implementing dual career guidelines at HEIs, it is crucial to identify relevant dual career aspects that also feasible to be implemented at HEI level in relation to the needs of students-athletes as well as to the opinions of HEI experts who are aware of the mechanisms that regulate the university. Thus, the present work aimed to identify the convergence of the opinions of European HEI experts on possible implementation of the six dual career policies and provisions areas resulting from previous phases of the MTG project [32], in relation to the extant dual career policy adopted by their academic institutions. It was hypothesized that the opinions of European HEI experts collected as the last phase of a Delphi method based on qualitative research, quantitative, and mixed-methods could contribute to foresee implementations of dual career policies, services, and provisions at the European university level [27].

## Material and methods

### Experimental approach to the problem

All procedures involving human participants in this study were in accordance with the institution’s ethical standards, the 1964 Helsinki declaration, and its later amendments or comparable ethical standards. The present work involved experts in virtue of their training or expertise and academic position (i.e., administrative staff, manager of sports services, dean, vice dean, head of the department, professor, associate professor, lecturer, and researcher). Experts have information and knowledge in a substantive area beyond that of the average person and who regularly share this information and knowledge through consultation, teaching or public speaking, or publications and written reports. For the Committee of the Protection of Human Subject (CPHS) purposes, experts are not human subjects when asked to provide opinions within their areas of expertise and do not require CPHS approval. Furthermore, in the present work, the experts’ opinions were about the external topic (e.g., aspects deemed relevant for dual career policies and services), not including demographic queries about age, education, income, or other personal information. Participation in the task was voluntary, and written informed consent was assumed with participants’ replies that they were willing to participate. Participating experts were free to opt out anytime without providing any reason, and incomplete opinions were not considered. In the quest to ensure better knowledge on dual career of athletes at the HEI level, the rights and welfare of research participants have always been protected, and confidentiality has been ensured and maintained throughout the research. For the above reasons, IRB review was not required.

In the final phase of the Delphi process, an online survey was selected to allow time and geographic flexibility in addition to multimedia and self-administration [33]. Thus, a bivariate go-zone plot was used to show the relationship between the mean ratings of the relevance and feasibility of the dual career aspects emerging from the previous two phases of the project. Finally, Qualitative Data Analysis method guided the development and implementation of this research [34].

### The instrument

According to the literature [32, 35], the conceptualization of the aspects that could facilitate the implementation of dual career at the HEIs was achieved in the previous two steps of this multistage Delphi method. The questionnaire encompassed two sections to gather information on: 1) the country, type of HEI (e.g., private or public), academic position (i.e., administrative staff, manager of sports services, dean, vice dean, head of the department, professor, associate professor, lecturer, and researcher), European Research Council (ERC) area of expertise (i.e., Social Sciences and Humanities, Physical Sciences and Engineering and Life Sciences) of the respondents and 2) the actual availability of dual career services in the respondents’ HEIs and the respondents’ opinion regarding the relevance of 26 individual dual career aspects and the feasibility of their implementation at HEI level utilizing a 10-point Likert-type scale (lowest value = 1; highest value = 10). As an example, in the financial support thematic area, “scholarship” is described as a grant or payment made to support a student-athletes’ education awarded on academic or/and sports merits. Specifically, the participants are requested to answer if this service is already in place at their university, and to rate its relevance and feasibility for dual career implementation. Furthermore, for each of the proposed dual career aspects, respondents were allowed non-mandatory open-ended questions (e.g., in your opinion, can it be implemented and how?) for further suggestions (S1 File). No personal sensible information was collected that could identify the participants.

### Recruitment

To ensure an appropriate representation of HEI experts, a purposeful sampling was deemed suitable and a core strength to gain a comprehensive, meaningful, and practical knowledge of dual career at the university level. By screening their national and European networks, the MTG team identified and invited potential participants and informed them that their contribution was voluntary and anonymous and that they could withdraw from the study anytime without giving any reason. Thus, a link to the online survey was provided and informed consent was assumed from completing the survey. On March 1, 2020, an official invitation was sent to the participants with a 7-day follow-up. The deadline for data collection was May 14, 2020. This procedure was deemed necessary to increase the response rate for the online surveys encompassing more than 20 items [33].

### Data analysis

According to the literature [36], the mean ratings of the relevance (x-axis) and feasibility (y-axis) for the identified dual career aspects were used to plot the position of each aspect relative to all the other aspects. The resulting scatterplots identified four quadrants (i.e., I. II. III. and IV) of go-zone, reporting aspects deemed feasible but considered to have a low relevance (Quadrant I), aspects that have been attributed low ratings for both relevance and feasibility (Quadrant II), aspects deemed relevant but considered to have low feasibility (Quadrant III), and aspects deemed to be most feasible and having the highest relevance (Quadrant IV), respectively. In particular, Quadrant IV identified the aspects HEI experts rated as highly relevant for feasible implementation of dual career programmes at the university level. Data were analysed by means of the Statistical Package for the Social Sciences version 24.0 (SPSS Inc., Chicago, Illinois). Descriptive statistics encompassed frequency of occurrences and percentages for dual career services, whereas means and standard deviations were calculated for the ratings.

For the qualitative data analysis, two independent researchers read several times the participants’ responses to the open-ended questions to become familiar with the content and elaborate on themes associated with the purpose of implementing dual career at HEIs. Then, the researchers independently created a series of categories for each question and individually positioned each response in one or more categories before comparing their respective evaluations to reach an agreement [37]. Any uncertainty and disagreement were resolved by consulting a third researcher of the MTG Team.

## Results

### Characteristics of HEI experts’ population

The survey involved 71 HEI experts from 12 Member states (i.e., Croatia, Denmark, Ireland, Italy, Latvia, Poland, Portugal, Romania, Serbia, Spain, Sweden, and Switzerland). Empty questionnaires and duplicates were removed. The majority of the respondents were university professors (40%), sports office managers (11%), researchers (11%), heads of department (8%) and course of study (9%), lecturers (8%), vice-deans (8%), deans (5%), and administrative-technical staff employers (2%). In addition, the respondents were involved in Life Sciences (88.5%), Social Sciences and Humanities (9.8%), and Physical Sciences and Engineering (1.6%), respectively.

### Dual career services at higher education institutes

Interviewees declared their inability to respond in 14.9 ± 6.8% of the cases, with the highest rate relating to their awareness of national dual career policies (31%). Concerning the six categories of the identified 26 dual career aspects (Table 1), Logistic Support (62.3%), Assistance/Tutorship (40.6%), and Curricula Requirements (40.1%) resulted the most available services. In contrast, Social Support (29%) and Financial Support (29%) were the least present services. Of the 26 identified dual career aspects, only 39.3 ± 16.0% were already present at the HEIs level, even if most respondents considered them implementable (Table 2). The most frequently extant provision is the access to educational facilities, whereas the presence of an institutional dual career committee was the least represented. Furthermore, in three cases, respondents left questions unanswered.

**Table 1:** Extant dual career policies and provisions at higher education institutes in descending order.

Category	Present (%)	Not present (%)	Don’t know (%)	No answer (%)
Logistic Support	62.3	27.8	9.9	0.0
Assistance/Tutorship	40.6	43.2	15.7	0.5
Curricula Requirements	40.1	46.1	13.7	0.0
Financial Support	36.6	53.2	10.2	0.0
Social Support	29.3	53.3	16.9	0.5
Policies	28.6	46.9	23.9	0.5

**Table 2:** Frequency of occurrence of dual career services for student-athletes at the higher education institutes.

Category	Aspects	Present (n)	Not present (n)	Don’t know (n)	Implementable (n)	No answer (n)
Assistance/tutorship	DC proactive programmes (capable to act autonomously, even anticipating needs)	22	29	19	48	1
	DC programmes based on individuality (adaptable to individual needs)	31	28	11	50	1
	DC programmes based on the integration of cooperation between academic departments, sports or professional services	24	33	14	53	0
	Psychological support	24	41	6	58	0
	Tutorship/Mentorship	41	23	7	60	0
Curricula requirements	Distance learning	39	27	5	55	0
	Individualized study plan	31	31	9	52	0
	Recognition of ECTS for the sport career	26	36	9	48	0
	Untraditional learning strategies (e.g., creating digital portfolios, using social networks)	18	37	16	50	0
Financial support	Remission of tuition fees for S-As	25	38	8	51	0
	Salary	22	41	8	45	0
	Scholarships for S-As	32	33	6	60	0
	Other (financial support)	25	39	7	49	0
Logistic support	Access to educational facilities (e.g. gymnasium, internet, e-mail services, e-libraries, labs, research centers)	65	2	4	63	0
	Accommodation facilities for S-As	29	37	5	49	0
	Economic investment for university facilities	40	19	12	58	0
	Sport facilities	43	21	7	53	0
Social support	Institutional DC committee	10	49	12	56	0
	Local to international seminars, workshops, and meetings on up-to-date DC issues	32	26	13	60	0
	Peer to peer support	12	39	19	51	1
	Publicity for S-As representing university	34	29	7	60	1
	Publicity on the S-As and their characteristics suitable for the labour market	20	36	15	55	0
	Seminars, workshops, meetings with parents and coaches	17	48	6	47	0
Policies	National DC policies	25	24	22	55	0
	Special access contingent (reserved for actual or ex high sport performance practitioners)	22	35	14	39	0
	Sport observatory of the university (controlling and monitoring the application of the DC statute)	14	41	15	55	1

n = Frequency of occurrence; S-As = Student-athletes; DC = dual career.

### Go-zone

**Fig 1:**
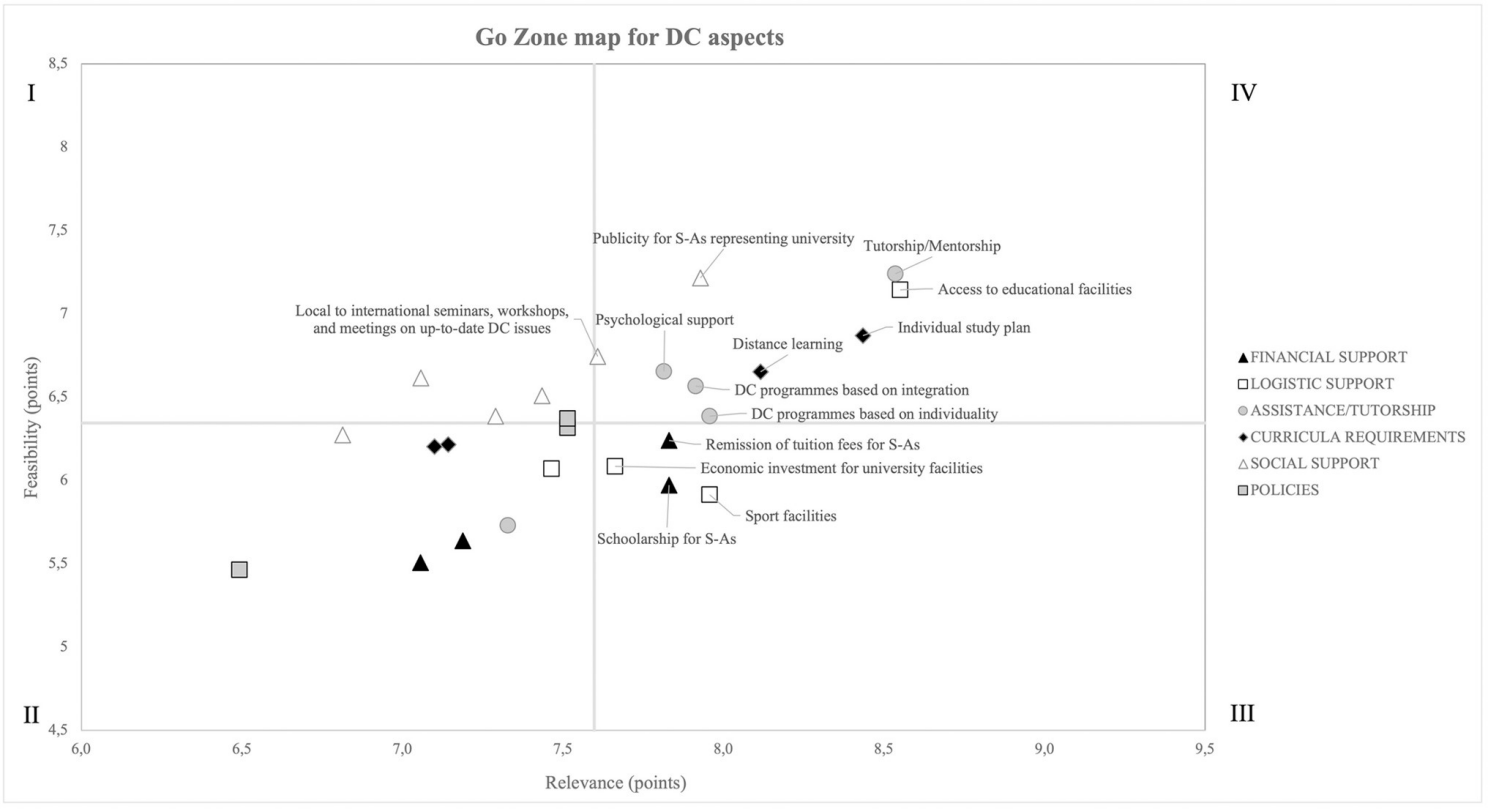
Go zone map of relevance and feasibility of implementation of dual career aspects. Quadrant I includes aspects deemed feasible but considered to have a low relevance; Quadrant II includes aspects with low relevance and feasibility; Quadrant III includes aspects deemed relevant but considered to have low feasibility; Quadrant IV includes aspects deemed both feasible and with high relevance.

Overall, higher values emerged for relevance (7.6 ± 0.5 points) with respect to feasibility for implementation (6.5 ± 0.5 points). Of the ten dual career aspects showing the highest ratings for relevance (Table 3), nine were included in Quadrant IV (Fig 1), four belonging to the category of Assistance/Tutorship (i.e., tutorship/mentorship; psychological support; dual career programmes based on integration of academic departments and sports services; and dual career programmes able to adapt to the specific needs of each S-A), two related to the Curricula Requirements (i.e., individual study plans; and distance learning), two were included in the Social Support category (i.e., publicity for student-athletes on the university webpage and initiatives for increasing the awareness of student-athletes and knowledge of dual career issues through seminars, workshops, and meetings, the realization of a student-athletes hall of fame, etc.), and one to the Logistic Support (i.e., access to educational facilities), respectively. Whilst two aspects included in the categories Financial Support (e.g., scholarships and tuition fees remission) and Logistic Support (i.e., investments in university and sports facilities) were considered relevant but less implementable (e.g., Quadrants III), the three aspects relative to the Policy category were included only in Quadrants I and II.

**Table 3:** Aspects considered relevant for dual career in descending order.

Aspect	Category	Quadrant	Relevance (points)	Feasibility (points)
Access to educational facilities (e.g., gymnasium, internet, e-mail services, e-libraries, labs, research centers)	Logistic support	IV	8.6 ± 2.3	7.1 ± 2.4
Tutorship/mentorship	Assistance/Tutorship	IV	8.5 ± 2.2	7.2 ± 2.1
Individual study plan	Curricula requirements	IV	8.4 ± 2.6	6.9 ± 2.6
Distance learning	Curricula requirements	IV	8.1 ± 2.4	6.7 ± 2.8
Sport facilities	Logistic support	III	8.0 ± 1.6	5.9 ± 2.9
DC programmes based on individuality (adaptable to individual needs)	Assistance/tutorship	IV	8.0 ± 2.1	6.4 ± 2.6
Publicity for S-As representing university	Social support	IV	7.9 ± 2.4	7.2 ± 2.4
DC programmes based on integration (unifying academic department. sports or professional services)	Assistance/tutorship	IV	7.9 ± 1.7	6.6 ± 2.3
Schoolarship for S-As	Financial support	III	7.8 ± 2.3	6.0 ± 2.6
Remission of tuition fees for S-As	Financial support	III	7.8 ± 2.3	6.2 ± 2.6
Psychological support	Assistance/tutorship	IV	7.8 ± 2.0	6.7 ± 2.4
Economic investment for university facilities	Logistic support	III	7.7 ± 2.4	6.1 ± 2.4
Local to international seminars, workshops, and meetings on up-to-date DC issues	Social support	IV	7.6 ± 2.6	6.7 ± 2.1
National DC policies	Policies	II	7.5 ± 2.5	6.3 ± 2.7
Sport observatory of the university (controlling and monitoring the application of DC statute)	Policies	II	7.5 ± 2.2	6.4 ± 2.4
Accomodation facilites for S-As	Logistic support	II	7.5 ± 2.6	6.1 ± 2.6
Institutional DC committee	Social support	I	7.4 ± 2.5	6.5 ± 2.5
DC proactive programmes (capable to act autonomously, even anticipating needs)	Assistance/tutorship	II	7.3 ± 2.3	5.7 ± 2.4
Publicity on the S-As and their charcteristics suitable for labour market	Social support	I	7.3 ± 3.1	6.4 ± 2.7
Other (financial support)	Financial support	II	7.2 ± 2.4	5.6 ± 2.5
Recognition of the ECTS for the sport career	Curricula requirements	II	7.1 ± 2.6	6.2 ± 2.7
Untraditional learning strategies (e.g., crating digital portfolios, using social networks)	Curricula requirements	II	7.1 ± 2.8	6.2 ± 2.5
Peer to peer support	Social support	I	7.1 ± 2.7	6.6 ± 2.5
Salary	Financial support	II	7.1 ± 2.8	5.5 ± 2.6
Seminars, workshops, meetings with parents and coaches	Social support	II	6.8 ± 2.6	6.3 ± 2.9
Special access contingent (reserved for actual or ex high sport performance practitioners)	Policies	I	6.5 ± 3.3	5.5 ± 2.8

### Open-ended questions

S1 Table in S1 File summarizes the open answers of respondents and provides examples of the suggestions. In relation to the Assistance/Tutorship category, HEI experts identified professors or dedicated dual career staff as potential tutors and envisaged a dedicated dual career office, involving also actual and former student-athletes as mentors. In addition, sports psychologists should offer psychological support for student-athletes in a dedicated office. Furthermore, courses and seminars on psychological challenges faced by student-athletes have also been suggested. Conversely, one respondent claimed that dual career is not of interest for sports psychologists.

The experts deemed necessary all the aspects included in the Curricula Requirements category. Whilst the implementation of individual study plans needs elective courses, a tutorship programme, or a written agreement between student-athletes and university and interactions with sports bodies, distance learning rely more on the availability of information technology (IT) services, depending on the subject (theory vs. laboratory). The recognition of academic credits for sports achievements (e.g., a call-up to the national team, sports qualifications and results) or soft skills based on a conversion table to be established within the European Credit Transfer and accumulation System (ECTS) [38] were also envisaged. Furthermore, social networks, dedicated software/webinars, and digital portfolios have been suggested as instruments/strategies for the adoption of untraditional learning strategies, as well as the recognition of soft skills and mentoring.

To provide Financial Support to student-athletes, a collaboration between HEIs, sports bodies (e.g., sports federations, clubs, and other actors of the sports system), and governments (e.g., sports and education ministries) has been envisaged. In particular, universities and sports bodies should provide scholarships, vouchers for meals, travel, services (e.g., physiotherapy), awards, and salaries. Furthermore, based on sporting merit or personal income of the student-athletes, HEIs and governments are recommended to allow tuition fees remission or fee reduction, whereas sports clubs/federations, HEIs, and governments are expected to provide a salary.

Concerning Logistic Support, accommodation, and sports facilities in or close to campus are envisaged, possibly free for student-athletes, especially if not on campus. On the other hand, educational facilities should be implemented mainly in terms of IT services. Further Logistic Support should include several infrastructures such as libraries, guesthouses, and cultural centres or transportation (e.g., car or transport service). In terms of investment, improvement of existing infrastructures or building new ones is considered a priority, followed by investments for improving transportation, management, and research projects.

Regarding Social Support to student-athletes, the HEI experts consider the academic staff, the student community, and the sports federation the main recipients of local to international meetings, workshops, and seminars (also online) also directed to stakeholders/employers and local and national governments. On the other hand, meetings, workshops, and seminars with family members and coaches should be performed regularly, also informally, with family and team members together or not, depending on the situation. Furthermore, it has been suggested that individual consultation with family members and coaches could be more useful to inform on individual requirements and career paths, also involving teams and federations. Press office and interviews, social media, and web pages are needed for publicizing student-athletes representing the university. Also, publicity for university games, the inclusion of the student-athletes in the university hall of fame, and a marketing office/sponsorship have been suggested. To facilitate the transition of student-athletes to the labour market, letters of recommendation, internships, business collaboration with companies and federations have been suggested, together with advertising on dedicated web pages and social media. The institution of a dual career committee should be included in the university regulation, depending on academic decision makers, and working tables, developed at various levels, and connected to the development of a tutorship programme. The implementation of peer-to-peer support to be recognized through awards should be based on sports merit boards, hall of fame, web pages, and ECTS.

For the Policy category, the HEI experts consider the national legislation crucial to increase the awareness on dual career and involve sports and education ministries as well as the council of rectors. In adhering to the European guidelines and encompassing both high-level and sub-level athletes, national legislation is deemed important to ensure homogeneity between all universities. Web pages, hall of fame, and tutorship programmes play a central role also in the institution of a sports observatory responsible for controlling and monitoring the application of the dual career policy of student-athletes of the University. However, the implementation of existing observatories is also suggested as a strategy. Furthermore, agreements among universities, the creation of a national database, scientific research, and surveys have been suggested as applicable. Finally, special entry requirements should be reserved not only for actual and former high-level athletes, but also for all the athletes and artists should be considered in the national legislation and achieved by employing dedicated programmes/webpages and mass media.

## Discussion

Previous studies investigated the subjective perception of student-athletes regarding their dual career barriers and services at university level [16, 39]. Undoubtedly, being the final recipients of policies and services for dual career, the student-athletes’ point of view is crucial for recognizing problems and foreseeing solutions for building a dual career-supporting environment. However, student-athletes may be unaware of the challenges and costs for implementing the required dual career policies and provisions at HEI level. Therefore, the findings of the present study bridge the gap between the needs of university student-athletes [32], and a reasonable implementation of dual career services. Based on thorough consultations with European university student-athletes [32, 35], the last step of the Delphi survey technique used in this study resulted suitable for exploring the possible implementation of dual career at tertiary education according to the opinions of European HEI experts with varying backgrounds, necessary to guide the actual implementation of dual career services at academic level. In particular, the organization of the HEI experts’ responses into four quadrants of the go-zone graph allowed identifying alternative objectives for creating strategies to allocate resources between different options efficiently. Thus, the present findings revealed novel and different aspects of the complex implementation of dual career policies and provisions and represent a starting point for initial tentative speculations to be corroborated by future research in this area.

Nine aspects belonging to the Assistance/Tutorship, Curricula Requirements, Logistic Support, and Social Support categories have been considered both relevant and feasible for advancing dual career programmes at HEIs, which should be prioritized by academic policy makers. The four aspects belonging to the logistic support and financial support categories in the quadrant III are deemed important to develop a dual career-supporting environment but less feasible due to foreseen difficulties in their implementation. Further insights derive from the HEI experts’ answers to the open-ended questions, which have been orgnanized insix dual career categories.

### Assistance/Tutorship

At the meso dimension of dual career, individuals having strong, direct, and personal relationships with the athlete in the family (e.g., parents, siblings, relatives, friends, and peers), the sport (e.g., coaches, managers, staff, dual-career tutor), and the academic (e.g., classmates, teachers, tutors, deans) environments play a crucial supporting role of the student-athletes entourage [40]. Coherently, in the present study assistance and tutorship for student-athletes has been most regarded as relevant and implementable service at HEI level. These findings substantiate the crucial role of tutors and counsellors with a thorough understanding of dual career challenges and opportunities as valuable supporters of the academic success of student-athletes [7–9, 23, 25, 41, 42]. In general, respondents declared that dual career tutorship/mentorship is the only service offered at their HEIs. In considering that HEIs usually provide several services for students (e.g., academic orientation, consultation, career and personal counselling, and psychological support), it seems quite feasible to prioritize the implementation of dual career by providing specific training to the HEI personnel, also considering possible connection between different actors of the meso dimension such as parents and coaches [6, 43–46]. In addition to psychologists and dual career staff, HEI experts also identified professors having a positive perception of student-athletes as potential tutors to assist the sport-study combination and to create an academic environment promoting the student-athletes commitment to higher education [47, 48].

Even though empathic attitudes of well-informed service providers and faculty members are envisaged for enhancing positive academic experiences for student-athletes and building a university readiness mindset, HEIs are called to develop/implement dual career programmes and dedicated services to ensure interdepartmental collaboration and coordination, which are crucial for promoting tailor-made arrangements and committed guidance to sustain student-athletes [7, 47, 49]. The HEI experts also deemed relevant pro-active dual career programmes potentially able to anticipate the student-athletes needs and/or challenges; however, they diagnosed them with low feasibility. Indeed, pro-active programmes could be feasible if based on high-quality practices and extensive experience in the student-athletes needs [49, 50]. However, in many Member States, dual career is still in its infancy stage and these findings corroborate the need to integrate efforts through further intra- and inter-country exchanges of best practices to build a solid European dual career discourse [7, 42, 51].

### Curricula requirements

When designing programmes to enhance academic success, arrangements should be attuned to specific student populations [50]. In particular, HEI experts considered individualized study plans and distance learning aspects as high priorities with a high potential for implementation. Apart from distance learning massively implemented during the pandemic lockdowns [52, 53], most respondents declared a lack of individualized study plans in their institutions (Table 2). Actually, athletes engage in various sports under different competitions and training schedules taking place in unique facilities and venues. Therefore, not only HEI experts indicated individualization of study plans as a critical aspect, but they also underlined that it needs to be supported by a tutorship programme helping student-athletes developing connections to the institution and providing an orientation to campus resources and services, as well as encompassing elective courses aiming to enhance concentration, relaxation, psychological well-being, and self-efficacy for improving academic readiness [15, 54]. Furthermore, written agreements between the student-athlete and the university have been suggested to ensure the proper implementation of individualized study plans. Agreements could also profit from a co-construction process involving sports bodies to verify the compatibility of potential conflicting commitments and to increase the likelihood of meaningful learning situations for athletes persisting towards their degree completion, particularly relevant for the first-year student-athletes who might undergo various academic and social changes [49, 55].

Traditionally, academic culture has been reluctant to adopt technological teaching practices due to teachers and students’ lack of proper organizational support and/or digital literacy [56]. Coherently, the HEI experts considered innovative teaching and ECTS recognition for informal and non-formal learning through sports participation less feasible with respect to their relevance. Actually, distance learning was crucial during the COVID-19 pandemic lockdown, which imposed HEIs on a rapid change towards the online delivery mode of teaching utilizing synchronous and asynchronous classes, webinars, social networks, and distance evaluation [52, 53, 57, 58]. Thus, an online scheduled curriculum-based study could be maintained to guarantee adequate support to student-athletes, who could benefit from online-based study plans when unable to attend onsite classes and examinations [52]. Furthermore, to represent the student-athletes accomplished work and to extend beyond initial degree conferment, HEI experts suggested the adoption of digital portfolios that could foster the students’ critical thinking and equip them with a website for post-graduation employment searches [59]. In fact, athletes could face some difficulties to enter the labour marked when their sports career comes to an end, due missed social and work opportunities during their sport commitments. For this reason, helping student-athletes building their post-sport careers is crucial; indeed, the creation of a personal portfolio could be very valuable [60]. Finally, in considering that the transfer of skills and knowledge are core concepts in the education and employment policy discourse to face the actual and future challenges in technologies, markets, and organizations [61], HEIs are urged to establish the ECTS conversion system encompassing the valuable soft skills developed in and through sports (e.g., commitment, teamwork, respect, goal orientation, self-efficacy, time management, responsibility, and autonomy, etc.), which proved to have positive effects on career success and labour market trajectories of athletes [62, 63].

### Financial support

To attend tertiary education, European students have to sustain different financial burdens in relation to the policies adopted by the Member States and the HEI typology (e.g., public or private). In general, the European approach to tertiary education considers HEIs as a collective good, with students benefiting from full or partial state support and having no or low tuition fees. On the other hand, if HEIs are intended as a private investment, students must sustain high tuition fees unless benefiting from a subsidy regimen, which could prevent long-term individual debt. Moreover, Whist the European separation between academic and sports systems likely resolves in athletes being influenced by the sport context outside the university, private universities are more likely to support the elite sport financially as a marketing strategy to attract media attention and prospective students [64–66]. Thus, along a continuum, HEIs’ regimes could be categorized as low-tuition–low-subsidy, low-tuition–high-subsidy, or high-tuition–high-subsidy [67].

Due to the global economic crisis beginning in 2008 and the rapidly changing economies, significant reductions in government funding to HEIs shifted the cost of higher education from the state to the individual student, whereas others continued to support universities as critical institutions but with reduced budgets [68]. Although the present results indicate that some forms of financial support for student-athletes are already present in some HEIs (Table 2), it is not surprising that the respondents considered these aspects relevant but challenging to implement, allocating the remission of tuition fees and scholarship in Quadrant III. Consequently, the HEI experts envisaged a collaboration between HEIs, sports bodies and public sectors to overcome the limited funding and reduce the financial pressures for the cost of higher education (e.g., tuition fees, books, housing and food, tutor, etc.) and sport (e.g., equipment, training camp, physiotherapy, travel, etc.), which might determine dual career dropouts of student-athletes [46, 69–71].

Despite many athletes compete in non-revenue-generating sports, and very few elite student-athletes are financially independent through their sports earnings, they could generate substantial value for their HEIs and sports bodies, especially when engaging followers through social media platforms [72, 73]. Thus, independently of the private or public nature of HEIs, student-athletes could be popular choices to target for brand endorsement opportunities and to align internal and external dimensions achieving collaborative practices to co-create sport-related values [73–76].

### Logistic support

Overall, the Logistic Support resulted in the most present provision for university student-athletes. The access to educational facilities resulted provided by almost all the experts’ institutions and embodied in Quadrant IV, whereas sports facilities and economical investment for university facilities are included in Quadrant III and less implemented (Table 2, Fig 1). Conversely, accommodation facilities for student-athletes were present in less than the half of the experts’ HEIs (Table 2) and were located in Quadrant II, thus considered less relevant and implementable (Fig 1). Along with the natural focus on educational facilities (e.g., study halls/study tables, libraries, computer halls, etc.), HEIs’ vision and values grounded in the holistic education of students highly consider physical activity and sports participation in a prime position to promote the development of crucial skills consistent with educational values such as health habits, social relations, and goal setting, which are likely to be maintained in later years [77]. However, besides being governed by the principle of autonomy, extracurricular sports programmes at universities vary, depending both politically and financially on resources.

In general, to serve the broad student community, HEIs are equipped with multi-purpose gymnasia, pitches, and swimming pools. However, since elite sports greatly depend on specific equipment, facilities, and/or environmental conditions (e.g., winter or sea sports), university resources unlikely respond to the needs of the various elite sport domain. Thus, HEIs must establish positive relationships, especially with sports federations with high-performance facilities in their neighbourhood, which could also help accommodate student-athletes to enhance the incoming relocation and/or migration of elite student-athletes [78]. In fact, regarding student accommodation, it is quite unlikely to foresee university housing reserved for student-athletes because, in Europe, only an average of 18% of students live in student accommodations, particularly international students and students who depend on national public student support [79]. Furthermore, since student-athletes spend a quite amount of time transferring each way from home to the university and the training venues, HEIs are urged to consider the logistics by facilitating efficient academic schedules or establishing specific arrangements for transportation [7, 72]. Additionally, concern has recently been raised about whether athletes spend too much time in sedentary behaviour off-training [80]. Therefore, optimizing logistic support to encourage the adoption of active lifestyles throughout the day and limiting sedentary opportunities as much as possible in specific domains such as transport and leisure time is vital for the student-athletes’ well-being who already spend a long-time sitting studying [52].

### Social support

Students are expected to establish positive relationships with faculty and peers to build a sense of confidence, a focus on academic performance, feelings of support, and encouragement leading to the achievement of a university degree and solid bonds to the university community [81]. In being involved in different activities at different times, university students benefit from their academic experience, with social integration, classroom interactions, interactions with faculty, and activities enhancing personal development, learning, and cognitive growth. Indeed, HEIs have the responsibility to allocate resources for learning opportunities, student engagement programmes, and services that foster student involvement [82]. Due to demanding sports responsibilities, student-athletes often have limited time to engage in the university level’s social experience, which might affect their feeling of belonging or connection to faculties and peers [72]. In the present work, Social Support for student-athletes was considered relevant and highly implementable (e.g., Quadrant IV). These findings indicate that HEI experts consider student-athletes a resource for drawing national visibility to the universities, thus attracting interest from alumni, legislators, and prospective students [15]. Actually, the significant amount of time and effort student-athletes invest into sport could disconnect them from their university peer groups, leading to feelings of isolation, separation, loneliness, and fatigue from a dual career commitment [47, 72]. At an institutional level, faculty and administration are urged to reconsider the importance of the peers’ community. They should reward classmates for showing cooperative behaviours by offering authentic interactions in support of student-athletes, thus favouring a positive educational climate and a sense of belonging, leading to a unique and significant educational path towards degree completion on time. Indeed, the cooperation between student-athletes and academic role-set members (e.g., professors and non-athlete students) could be enhanced by establishing a dual career committee, which could be an integral part of the day-to-day functioning of the various stakeholders within and beyond the HEIs. In fact, HEIs base their internal quality assurance on committees encompassing an active involvement of staff and students deemed crucial to build a quality culture through sharing information for self-assessment [83]. To enhance their quality assurance process, HEIs should also consider a committee effectively dedicated to student-athletes as atypical students and connected to developing a tutorship programme. In particular, the dual career committee should track the dual career support systems and the student-athletes’ academic experience so that support may be adjusted as required.

In addition to the relevant role sports participation has on health, social welfare, and the economic benefits for European society, athletes can be considered valuable human capital for the labour market for their informal education acquired in and through sports, which helps them to develop skills directly and/or indirectly impacting positively on labour market outcomes, job quality and earnings [84]. Thus, HEI experts are strongly advised to reconsider the relevance of creating the right environment and actions (e.g., collaborations with companies, letters of recommendation, internships) to fostering the employability of (former) elite athletes, which not only would align with the recommendations of the European dual career guidelines but also could invest in the institutional prestige and the quality of education delivered [9].

### Policies

The European Commission recommended the Member States to develop national dual career guidelines concerning their country-related specificity of sport and education systems and cultural diversity [9]. Indeed, policies clarify the roles of various dual career stakeholders as well as their rights and obligations, which help defining expectations and planning for possible structural implementation through a thorough evaluation of actual strengths, weaknesses, opportunities, threats, and resources. However, at present national recommendations have been published only in Sweden, and few other countries regulate the principles of sport and education, whereas a systematic dual career-friendly approach is often lacking at the European higher education level [7, 85]. Although HEIs are recommended to include dual career within their strategies, in the present study, respondents reported a limited presence of dual career policies in their universities and assigned low relevance and feasibility for implementation values to the three policy-related aspects [8, 9]. These findings substantiate that HEI experts tend to underestimate the importance of a legal basis in dual career, thus overlooking its crucial role in ensuring transparency and equality in addition to raising cultural awareness of various social institutions [86, 87]. In fact, dual career policy development could kick-start the process of establishing agreements with responsive dual career actors (e.g., policymakers, representatives from various HEIs, and sports bodies) for the implementation of strategic initiatives, practices, and support systems facilitating the academic path of athletes, especially during temporary relocation due to an ever-growing globalized sport [8, 9, 78]. Furthermore, the converting of HEI policies into practice requires continual vigilance through the adoption of a monitoring system to evaluate the application of the dual career policy of the university, which fosters a reflective thinking capacity and allows to capitalize from constructive feedback on the outcomes for envisaging further refinements [7, 86]. Although the open answers of the experts presented the institution of a dual career observatory as a suggested HEI strategy, the assignment of this policy aspect to Quadrant I highlights the perception of experts that services requiring financial investment and the involvement of decision-makers are difficult to implement, despite their relevance.

The present findings indicate that few HEIs adopt academic-specific admission procedures for student-athletes, even though this aspect is considered the least relevant and feasible for implementation. However, a lack of a clear definition of eligible athletes between HEIs has been highlighted, which not only determines the unequal treatment of European elite student-athletes but also limits the possibility of gathering harmonized data between Member States [7, 8, 72]. In considering that in Australia [88], Canada [89], New Zealand [90], and the United States [91] the student-athlete status is clearly defined, European HEIs should try to overcome specific cultural/organizational regulations and identify structured cooperation for the dual career eligibility process, recognition, and service provision, which could implement the European strategies in education [3, 4].

## Conclusions

The university departments and institutions are immediately responsible for implementing dual career policies and provisions, which are essential for student-athletes to know what is expected of them and what support they can expect from their university towards completing a degree [9]. Based on the needs and expectations of European student-athletes, this study was conceived with the understanding that the existing dual career policies and support provisions at the HEI level need to be grounded in a concern of their relevance and feasibility for implementation [32]. Thus, this research matches the collaboration of researchers and practitioners to develop a consensus about problems and solutions. This approach is founded on a phenomenological lifeworld-led approach to ideas that can be used to inform practical directions in dual career HEI settings. In fact, the everyday experience of the participating experts was a coherent and useful starting point to develop a deep understanding of the considered 26 aspects encompassed in the six delineated dual career categories, intended not to form a checklist but to emphasize insights into features of the best dual career at the tertiary educational level. As such, the study is concerned towards ‘actions’ over the ‘research’ component and offers impactful suggestions for dual career implementation of the European HEIs strategic agenda. The Quadrant IV of the resulting scatterplots identified nine aspects rated as highly relevant and highly feasible for implementation such as tutorship/mentorship, psychological support, programmes based on integration of academic departments and sports services, and adaptable programmes to the needs of each student-athletes (assistance/tutorship area), individual study plans and distance learning (curricula requirements area), publicity for student-athletes and initiatives for increasing the awareness of student-athletes and knowledge of dual career issues (social support area), and access to educational facilities (logistic support area) and may represent a standard for dual career policies and provisions. Thus, the present findings emphasize the current quest for dual career progress and reinforce the need for a minimum standard for dual career policies and provisions [7–9]. In this respect, well-structured cooperation systems between HEIs and other dual career stakeholders could provide opportunities to strengthen the potential of the athletes of the future [92, 93]. Thus, the MTG project enabled successful cooperation between HEIs and real changes at the Italian and Romanian partner universities, which introduced dual career policies despite their strict and demanding academic procedures. Finally, the MTG team developed the Guidelines to Promote the Dual Career of Athletes-Students: Methodology for Universities, and the Guidelines to promote the dual career of athletes-students: a manual for authorities, which are practical and reusable resources for the practitioners available at the European ERASMUS+ platform (https://erasmus-plus.ec.europa.eu/projects/search/details/603346-EPP-1-2018-1-LV-SPO-SCP).

This study presents some limitations. First, the present results should be viewed within the limited sample that could not represent the whole European scenario, so that further research is needed to generalize the present experts’ views. It is worth noting that many of the respondents declared expertise in sports sciences, probably valuing the sport’s commitment differently from their counterparts in different areas of expertise. Therefore, future investigations are needed to corroborate the present findings. Furthermore, additional research is needed to explore the proactive role that policymakers at the governmental level play in implementing legal constraints, university administrative rules, and financial support favouring dual career policies and provisions across tertiary education for the benefit of the holistic development of athletes.

## Supporting information

S1 File This file contains all the supporting text and table. (DOCX)
