# Supplementary material for: Exploring dual career quality implementation at European higher education institutions: Insights from university experts
Source: PLoS One. 2022 Nov 30;17(11):e0277485. doi: 10.1371/journal.pone.0277485 (PMC9710783; doi:10.1371/journal.pone.0277485)
Supplement: S1 File — (DOCX) [file pone.0277485.s001.docx]

Supporting information

**Questionnaire for Dual Career HEIs’ experts**

Students-Athletes (SAs) are individuals who dedicate their efforts to succeed in both sports and education. Despite their success is valuable for the society and the institutions they represent, they might find themselves in the unfortunate situation of having to choose between study and sport, thus compromising their human right to an education (United Nations, 1948), as well as the possibility of a potential elite sport career.

The term dual career (DC) describes *“athletes that combine, without unreasonable personal effort, their sporting career with education and/or work in a flexible way through high-quality training in order to protect their moral, health, educational and professional interests without compromising either objective, with a particular focus on the continued formal education of young athletes (European Commission, 2012)”.* At present, Dual Career programs across the European Union are heterogeneous and in most of them are at an early stage.

The *<insert here project name>* project aims to create Dual Career Guidelines for higher education institutions (HEI) and to provide effective Dual Career methodology for HEI.

To achieve our objectives and support Student-Athletes in their Dual Career, your opinion is really important, specifically related to the following to Dual Career aspects:

- Financial support
- Logistic support
- Assistance
- Academic support
- Social support
- Other support

Please, in filling the questionnaire feel free to suggest anything you think valuable for the development of effective Dual Career programmes.

We are very grateful for your help!

The *<insert here project name>* consortium

**Financial support**

1. Scholarship (a grant or payment made to support a student-athletes’ education, awarded on the basis of academic or/and sport merit).
   1. It already exists yes no I don’t know

If not/you don’t know

- 1. In your opinion, can it be implemented? yes no
  2. How? (e.g. covered by universities or federations, shared expense)_________________________________________________________________________________________________________________________________

____________________________________________________________________

- 1. In your opinion, how much is relevant from 0 to 10? (0, not at all; 10 absolutely relevant)
  2. d. In your opinion, how much is feasible from 0 to 10? (0, not at all; 10 absolutely feasible)

1. Remission of tuition fees for S-As (the financial cost set by university to be paid by students for educational services are free or reduced for SAs).
   1. It already exists yes no I don’t know

If not/you don’t know

- 1. In your opinion, can it be implemented? yes no
  2. How?___________________________________________________________________________________________________________________________________

____________________________________________________________________

- 1. In your opinion, how much is relevant from 0 to 10? (0, not at all; 10 absolutely relevant)
  2. d. In your opinion, how much is feasible from 0 to 10? (0, not at all; 10 absolutely feasible)

1. Salary (a fixed regular sum paid as a salary or as expenses).
   1. It already exists yes no I don’t know

If not/you don’t know

- 1. In your opinion, can it be implemented? yes no
  2. How?___________________________________________________________________________________________________________________________________

____________________________________________________________________

- 1. In your opinion, how much is relevant from 0 to 10? (0, not at all; 10 absolutely relevant)
  2. d. In your opinion, how much is feasible from 0 to 10? (0, not at all; 10 absolutely feasible)

1. Other forms of financial support
   1. Are you aware of other forms of financial support? yes no I don’t know

If not/you don’t know

- 1. In your opinion, could there be other forms of financial support? yes no
  2. Which?__________________________________________________________________________________________________________________________________

____________________________________________________________________

- 1. In your opinion, how much is relevant from 0 to 10? (0, not at all; 10 absolutely relevant)
  2. d. In your opinion, how much is feasible from 0 to 10? (0, not at all; 10 absolutely feasible)

**Logistic support**

1. Accommodation facilities for SAs.
   1. They already exist yes no I don’t know

If not/you don’t know

- 1. In your opinion, can they be implemented? yes no
  2. How? (e.g. close to educational provision and/or sport facilities)_________________________________________________________________________________________________________________________________

____________________________________________________________________

- 1. In your opinion, how much is relevant from 0 to 10? (0, not at all; 10 absolutely relevant)
  2. d. In your opinion, how much is feasible from 0 to 10? (0, not at all; 10 absolutely feasible)

1. Sport facilities (the financial cost set by university to be paid by students for educational services are free or reduced for SAs).
   1. They already exist yes no I don’t know

If not/you don’t know

- 1. In your opinion, can they be implemented? yes no
  2. How? (e.g. inside university campus, close to the university campus) ____________________________________________________________________

____________________________________________________________________

- 1. In your opinion, how much is relevant from 0 to 10? (0, not at all; 10 absolutely relevant)
  2. d. In your opinion, how much is feasible from 0 to 10? (0, not at all; 10 absolutely feasible)

1. Access to educational facilities (e.g. gymnasium, internet, e-mail services, e-libraries, labs, research centers, cultural centers, students’ associations, canteen, career counselling)
   1. It already exists yes no I don’t know

If not/you don’t know

- 1. In your opinion, can it be implemented? yes no
  2. Do you want to suggest some other kind of facilities?________________________________________________________________________________________________________________________________

____________________________________________________________________

- 1. In your opinion, how much is relevant from 0 to 10? (0, not at all; 10 absolutely relevant)
  2. d. In your opinion, how much is feasible from 0 to 10? (0, not at all; 10 absolutely feasible)

1. Economic investment for university facilities.
   1. It already exists yes no I don’t know

If not/you don’t know

- 1. In your opinion, can it be improved? yes no
  2. How?_________________________________________________________________________________________________________________________________

____________________________________________________________________

- 1. In your opinion, how much is relevant from 0 to 10? (0, not at all; 10 absolutely relevant)
  2. d. In your opinion, how much is feasible from 0 to 10? (0, not at all; 10 absolutely feasible)

**Assistance/tutorship**

1. Tutorship/Mentorship (the figure and action of people whose objective is to support and help students in their academic life).
   1. They already exist yes no I don’t know

If not/you don’t know

- 1. In your opinion, can they be implemented? yes no
  2. How? (e.g. professors with former experience as athletes, dedicated job position)_________________________________________________________________________________________________________________________________

____________________________________________________________________

- 1. In your opinion, how much is relevant from 0 to 10? (0, not at all; 10 absolutely relevant)
  2. d. In your opinion, how much is feasible from 0 to 10? (0, not at all; 10 absolutely feasible)

1. Psychological support .
   1. They already exist yes no I don’t know

If not/you don’t know

- 1. In your opinion, can it be implemented? yes no
  2. How? (e.g. former students, students who are finishing the university) ____________________________________________________________________

____________________________________________________________________

____________________________________________________________________

1. These programmes are/should be based on integration (unification of the academic department, sports or professional services necessary for the SAs)?
   1. Integration already exists yes no I don’t know

If not/you don’t know

- 1. In your opinion, can they be implemented? yes no
  2. How?___________________________________________________________________________________________________________________________________

____________________________________________________________________

- 1. In your opinion, how much is relevant from 0 to 10? (0, not at all; 10 absolutely relevant)
  2. d. In your opinion, how much is feasible from 0 to 10? (0, not at all; 10 absolutely feasible)

1. These programmes are/should be based on individuality (the knowledge that each program must be structured according to the specific needs of each athlete)?
   1. Individuality already exists yes no I don’t know

If not/you don’t know

- 1. In your opinion, can it be implemented? yes no
  2. How?___________________________________________________________________________________________________________________________________

____________________________________________________________________

- 1. In your opinion, how much is relevant from 0 to 10? (0, not at all; 10 absolutely relevant)
  2. d. In your opinion, how much is feasible from 0 to 10? (0, not at all; 10 absolutely feasible)

1. These programmes are/should be based on proactivity (the capability of the programme to act autonomously, even anticipating future needs)?
   1. Proactivity already exists yes no I don’t know

If not/you don’t know

- 1. In your opinion, can it be implemented? yes no
  2. How?___________________________________________________________________________________________________________________________________

____________________________________________________________________

- 1. In your opinion, how much is relevant from 0 to 10? (0, not at all; 10 absolutely relevant)
  2. d. In your opinion, how much is feasible from 0 to 10? (0, not at all; 10 absolutely feasible)

**Curricula requirements**

1. Individual study plan (a written agreement between the SAs and the University on courses to be acquired in a given period of time, ).
   1. It already exists yes no I don’t know

If not/you don’t know

- 1. In your opinion, can it be implemented? yes no
  2. How? _________________________________________________________________

____________________________________________________________________

____________________________________________________________________

- 1. In your opinion, how much is relevant from 0 to 10? (0, not at all; 10 absolutely relevant)
  2. d. In your opinion, how much is feasible from 0 to 10? (0, not at all; 10 absolutely feasible)

1. Distance learning (e-learning).
   1. It already exists yes no I don’t know

If not/you don’t know

- 1. In your opinion, can it be implemented? yes no
  2. How? (e.g. computer rooms in training camps, recordings of curricular classes, videoconference)

____________________________________________________________________

____________________________________________________________________

____________________________________________________________________

- 1. In your opinion, how much is relevant from 0 to 10? (0, not at all; 10 absolutely relevant)
  2. d. In your opinion, how much is feasible from 0 to 10? (0, not at all; 10 absolutely feasible)

1. ECTS (recognition of soft-skills acquired with sport).
   1. It already exists yes no I don’t know

If not/you don’t know

- 1. In your opinion, can it be implemented? yes no
  2. How? (e.g. training camps, competitions)

____________________________________________________________________

____________________________________________________________________

____________________________________________________________________

- 1. In your opinion, how much is relevant from 0 to 10? (0, not at all; 10 absolutely relevant)
  2. d. In your opinion, how much is feasible from 0 to 10? (0, not at all; 10 absolutely feasible)

1. Untraditional learning strategies t(e.g. develop a digital portfolio).
   1. It already exists yes no I don’t know

If not/you don’t know

- 1. In your opinion, can it be implemented? yes no
  2. How? (e.g. provision of dedicated software, social networks)

____________________________________________________________________

____________________________________________________________________

____________________________________________________________________

- 1. In your opinion, how much is relevant from 0 to 10? (0, not at all; 10 absolutely relevant)
  2. d. In your opinion, how much is feasible from 0 to 10? (0, not at all; 10 absolutely feasible)

**Social support**

1. Planned seminars, workshops and meetings during which DC information collected at institutional, local, regional, national and international levels should be provided.
   1. It already exists yes no I don’t know

If not/you don’t know

- 1. In your opinion, can it be implemented? yes no
  2. How? (e.g. for the academic staff, student community, federations)

____________________________________________________________________

____________________________________________________________________

____________________________________________________________________

- 1. In your opinion, how much is relevant from 0 to 10? (0, not at all; 10 absolutely relevant)
  2. d. In your opinion, how much is feasible from 0 to 10? (0, not at all; 10 absolutely feasible)

1. Planned seminars, workshops and meetings with all the family members and coaches.
   1. It already exists yes no I don’t know

If not/you don’t know

- 1. In your opinion, can it be implemented? yes no
  2. How?

____________________________________________________________________

____________________________________________________________________

____________________________________________________________________

- 1. In your opinion, how much is relevant from 0 to 10? (0, not at all; 10 absolutely relevant)
  2. d. In your opinion, how much is feasible from 0 to 10? (0, not at all; 10 absolutely feasible)

1. Assurance of wide publicity to student-athletes representing the University.
   1. It already exists yes no I don’t know

If not/you don’t know

- 1. In your opinion, can it be implemented? yes no
  2. How?

____________________________________________________________________

____________________________________________________________________

____________________________________________________________________

- 1. In your opinion, how much is relevant from 0 to 10? (0, not at all; 10 absolutely relevant)
  2. d. In your opinion, how much is feasible from 0 to 10? (0, not at all; 10 absolutely feasible)

1. Ensured wide publicity on the student-athletes and their characteristics suitable for the labour market.
   1. It already exists yes no I don’t know

If not/you don’t know

- 1. In your opinion, can it be implemented? yes no
  2. How?

____________________________________________________________________

____________________________________________________________________

____________________________________________________________________

- 1. In your opinion, how much is relevant from 0 to 10? (0, not at all; 10 absolutely relevant)
  2. d. In your opinion, how much is feasible from 0 to 10? (0, not at all; 10 absolutely feasible)

1. Establishment of an Institutional dual career committee, including representatives of the relevant actors of the athletes’ supportive entourage (e.g., athletes, family members, academic staff, sport staff, career staff).
   1. It already exists yes no I don’t know

If not/you don’t know

- 1. In your opinion, can it be implemented? yes no
  2. How?

____________________________________________________________________

____________________________________________________________________

____________________________________________________________________

- 1. In your opinion, how much is relevant from 0 to 10? (0, not at all; 10 absolutely relevant)
  2. d. In your opinion, how much is feasible from 0 to 10? (0, not at all; 10 absolutely feasible)

1. Envisaged peer-to-peer support to be recognized through awards, ECTS recognition, etc.
   1. It already exists yes no I don’t know

If not/you don’t know

- 1. In your opinion, can it be implemented? yes no
  2. How (e.g. hall of fame, sports merit Board, dedicated web page)?

____________________________________________________________________

____________________________________________________________________

____________________________________________________________________

- 1. In your opinion, how much is relevant from 0 to 10? (0, not at all; 10 absolutely relevant)
  2. d. In your opinion, how much is feasible from 0 to 10? (0, not at all; 10 absolutely feasible)

**Other**

1. National Legislation Governing the Status of the SAs.
   1. It already exists yes no I don’t know

If not/you don’t know

- 1. In your opinion, can it be implemented? yes no
  2. How (e.g. hall of fame, dedicated web page)?

____________________________________________________________________

____________________________________________________________________

____________________________________________________________________

- 1. In your opinion, how much is relevant from 0 to 10? (0, not at all; 10 absolutely relevant)
  2. d. In your opinion, how much is feasible from 0 to 10? (0, not at all; 10 absolutely feasible)

1. Sports Observatory of the University (This observatory is responsible for controlling and monitoring the application of the Statute of SAs of the University).
   1. It already exists yes no I don’t know

If not/you don’t know

- 1. In your opinion, can it be implemented? yes no
  2. How (e.g. hall of fame, dedicated web page)?

____________________________________________________________________

____________________________________________________________________

____________________________________________________________________

- 1. In your opinion, how much is relevant from 0 to 10? (0, not at all; 10 absolutely relevant)
  2. d. In your opinion, how much is feasible from 0 to 10? (0, not at all; 10 absolutely feasible)

1. Special access contingent (Each year, in the first phase of the national competition, the vacancies fixed for each course in each higher education institution are distributed by a general quota and by special quotas to which certain percentages of vacancies are reserved for actual or ex high sport performance practitioners).
   1. It already exists yes no I don’t know

If not/you don’t know

- 1. In your opinion, can it be implemented? yes no
  2. How (e.g. hall of fame, dedicated web page)?

____________________________________________________________________

____________________________________________________________________

____________________________________________________________________

- 1. In your opinion, how much is relevant from 0 to 10? (0, not at all; 10 absolutely relevant)
  2. d. In your opinion, how much is feasible from 0 to 10? (0, not at all; 10 absolutely feasible)

**S1 Table.** **HEIs’ responses to the open-ended questions.**

| **Category** | **Aspects** | **Grouped open-ended questions** | **Examples** | |
| --- | --- | --- | --- | --- |
| Assistance/  tutorship | Dual career proactive programmes (capable to act autonomously, even anticipating needs) | Monitoring | *"Based on their experience, dual career staff should anticipate the difficulties that the student-athlete will encounter, and put in place preventive and supportive actions before they occur.* | |
|  |  | Gap analysis/survey |  |  |
|  |  | Dedicated job positions |  |  |
|  | Dual career programmes based on individuality (adaptable to individual needs) | Through an *ad hoc* and individual study plan | *“To meet the needs of individualized dual career plans require resources for additional tutoring staff.”* | |
|  |  | Through individual consultation |  |  |
|  |  | Tutoring |  |  |
|  | Dual career programmes based on the integration of cooperation between academic departments, sports or professional services | Establishment of a sport/career center for guidance and counseling | *“Universities and clubs should work together, organize regular meetings, and create a specific department to deal with dual career issues.”*  *"Implementation could occur by specific dual career training for operators of existing student services."*  *“The student-athlete is not only a student, only an athlete, or only a student-athlete. He/she is a person with integrated social, family, academic, sport, citizenship needs. If one aspect fails, it can ruin all the other ones.”*  *“A good idea could be the organization of events during which athletes can make themselves known to the academic community.”* | |
|  |  | Establishment of a committee/dedicated office |  |  |
|  |  | Integration should involve external sports clubs and association within academic department and services |  |  |
|  |  | Through a mixed/concerted planning |  |  |
|  | Psychological support | Sport psychologist | *“Several Universities have their own psychologist, and he/she can be very helpful in the specific dual career area. Of course, dual career psychologic service could be more effective and successful if a sport psychologist is available or recruited.”* | |
|  |  | Psychologist (not sport psychologist) |  |  |
|  |  | Establishment of a dedicated office |  |  |
|  |  | Psychology courses |  |  |
|  | Tutorship/mentorship | Professor as a tutor | *“If the dean/rector supports student-athletes, mentorship within the campus can become a reality. Professors and/or former alumni elite athletes can be very useful to the current student-athletes. The sport staff has a special role in a mentorship process by providing feedbacks to all the dual career actors, and by helping student-athletes following their career goals.”* | |
|  |  | Student as a tutor |  |  |
|  |  | Establishment of a edicated office or job position |  |  |
|  |  | Dean delegate |  |  |
|  |  | Tutors from clubs (e.g., coaches) |  |  |
| Curricula requirements | Distance learning | Audio and video recording | *“To help student-athletes managing their study time when they are abroad for training camps or competitions, videoconferences, streaming or video lessons, and individual works could be effective. Also, classmates sharing notes/works can help the student-athletes’ learning and confidence.”* | |
|  |  | Computer rooms in training camps |  |  |
|  |  | IT services into the campus |  |  |
|  | Individualized study plan | Written agreement | *“Clubs/Sport bodies and universities should be aware of the importance of dual career and work together to create a coherent supportive environment for student-athletes.”* | |
|  |  | Tutorship |  |  |
|  |  | Elective courses |  |  |
|  |  | According to practiced sport |  |  |
|  |  | Through an interaction with the federation |  |  |
|  | Recognition of ECTS for the sport career | Based on sports results/qualification | *"The soft skills acquired through high-level national and international sports events should be recognized academic credits as elective activities to be included in the individualized study plan."* | |
|  |  | Based on national team call-up |  |  |
|  |  | Based on the recognition of the soft skills |  |  |
|  | Untraditional learning strategies (e.g., creating digital portfolios, using social networks) | Through the recognition of the soft skill | *“Non-traditional and innovative learning methods could be adopted to help student-athletes focusing on academic career. In fact, many athletes lack an academic focus and they need to spark their lives more.”* | |
|  |  | Digital portfolio |  |  |
|  |  | Implemented IT services |  |  |
|  |  | Implemented use of social networks for teaching |  |  |
| Financial support | Remission of tuition fees for student-athletes | Based on sporting merit and salary | *“Whilst private universities can easily implement financial help for student-athletes, public institutions encounter financial challenges. However, if a student-athlete faces financial difficulties, he/she can apply for the financial support provided to the students.”* | |
|  |  | University financial support |  |  |
|  |  | Financial support from external funds (e.g., government, sport system) |  |  |
|  |  | Partial remission of tuition fees |  |  |
|  | Salary | Private financing, sponsors, and volunteering | *“Financial support could be provided better by the sport sector. I wonder whether the educational institutions should have a role in providing a salary to student-athletes when other students do not receive it. I believe that club/sports federation should have a financial supportive role, whereas the universities could have an academic support.”* | |
|  |  | Salary provided by the university |  |  |
|  |  | Salary provided by the sports system |  |  |
|  |  | Salary provided by the government |  |  |
|  |  | Mixed fundings (e.g., university and sports system) |  |  |
|  |  | University team |  |  |
|  | Scholarships for student-athletes | Private financing, sponsors, and volunteering | *“To support sport-related expenses, my university of the student-athlete provides a 1000 euro-sport scholarships in addition to other financial dual career supports.”* | |
|  |  | Scholarship provided by the university |  |  |
|  |  | Scholarship provided by the sports system |  |  |
|  |  | Scholarship provided by the governement |  |  |
|  |  | Scholarship provided by co-fundings (e.g., university and sports system) |  |  |
|  | Other (financial support) | Investment for teching and training equipment | *“Student-athletes can be engaged for counselling services, training programs, sports demonstrations, etc. for other students under service contracts. These contracts could be stipulated in future regulations and could be achieved through university sports associations.”*  *“Free tuition fees to enroll to different courses in different faculties of our university.”*  *“Commuting expenses of student-athletes could be covered (e.g. money for fuel, public transport tickets).”* | |
|  |  | Post-career projects, research, services contracts |  |  |
|  |  | Vouchers for meals, travels, and services (e.g., physioteraphy, psychologist) |  |  |
|  |  | For migrant athletes, shared tuition fees between universities |  |  |
| Logistic support | Access to educational facilities (e.g. gymnasium, internet, e-mail services, e-libraries, labs, research centers) | IT services, research and cultural centers guesthouse | *“Internet services, e-mail services, e-libraries, laboratories, research centers, cultural centers, student associations, career counseling, sports facilities are free for all categories of students, including student-athletes. Student-athletes can be involved in demonstrations during physical activity lessons to other students. Elite student-athletes could be testimonial of the university, networking with other institutions, etc.”* | |
|  |  | Implementation of existing facilities |  |  |
|  | Accommodation facilities for student-athletes | Close to sport and education facilities | *"Support for short-term accommodation for student-athletes residing in other towns because of sporting activities. As an example, an accommodation for athletes who play for a club in a region far from that of their university having to sustain an exam in presence. However, for our experience, this service has never been requested."* | |
|  |  | Into the campus or very close |  |  |
|  |  | Temporary accommodation |  |  |
|  |  | Financed externally through sports system or sponsors |  |  |
|  | Economic investment for university facilities | Investment in facilities for all students | *“It can be difficult to invest in sport facilities. Some student-athletes need specific and expensive facilities that the University cannot afford. The relationship between recreational sport and elite training quality can challenge the Institution every day.”* | |
|  |  | Investment in facilities specific for student athletes |  |  |
|  |  | Investment in university-owned transportation for student-athletes |  |  |
|  | Sport facilities | In campus or close to campus facilities | *“Sport and training do not take place at universities in Denmark, but this could be a good idea - also with opportunities for reduced prices for dual career students.”* | |
|  |  | Free for student-athletes and with favored access |  |  |
| Social support | Institutional dual career committee | Dual career committee and focus group | *“Dual career committee to supervise the implementation, and to listen the student-athletes’ and teachers’ suggestions for implementation and improvement. This committee should include at least professors, students and sport staff.”*  *“Organization of dual career commttees at university, local (community level) or regional level.”*  *"A dual career reference person from various stakeholders at the educational, sports, family, labout market levels."* | |
|  |  | Multisectorial and multilevel committee |  |  |
|  | Local to international seminars, workshops, and meetings on up-to-date dual career issues | Dual career training for the academic staff |  | |
|  |  | Dual career training for the student community |  |  |
|  |  | Dual career training for federations and sports bodies |  |  |
|  |  | Dual career training for national and local government |  |  |
|  |  | Dual career training for a mixed audience |  |  |
|  |  | Dual career training by means of e-learning platforms |  |  |
|  | Peer to peer support | Hall of fame | “Any types of recognition. Hall of fame is a good idea, but, again, social media most probably. Maybe a mention in school's magazine/newspaper/journal and yearbook.” | |
|  |  | Sports merit board |  |  |
|  |  | Dedicated web page |  |  |
|  |  | Recognition on social media |  |  |
|  | Publicity for student-athletes representing university | Hall of fame | "Advertising the dual career programme mainly through social media and merchandising (e.g., student-athletes wearing University Dual Career gym clothes)" | |
|  |  | Dedicated web page and social media |  |  |
|  |  | Media coverage |  |  |
|  |  | Press office |  |  |
|  | Advertisement of the student-atletes and their characteristics suitable for the labour market | Advertisement through the placement platform and dedicated web pages and social media | *"Dedicated internships, meetings with companies, preparation of material to be distributed to companies with the characteristics of student-athletes to make them particularly interesting from a professional point of view."* | |
|  |  | Reinforcing business collaborations with the stakeholders |  |  |
|  |  | Letters of recommendation |  |  |
|  |  | Internships |  |  |
|  | Seminars, workshops, meetings with parents and coaches | Organization of regular dual career meetings | *“Coaches and family should be aware of the dual career path and their role in supporting the student-athlete. It would be sufficient to organize a meeting/workshop and/or provide a listening "center" and/or an online forum service."* |  |
|  |  | Individual and informal counselling organized on a regular basis with or without team members and staff |  |  |
| Policies | National dual career policies | Adherence to the guidelines | *"Definitely for elite student-athletes, but I believe also for sub-elite student-athletes who engage in demanding sport commitment (for example, at least 3-4 training sessions per week and regular championship/competitions), even regardless of outstanding athletic achievements."* | |
|  |  | Dual career policies for high-level athletes |  |  |
|  |  | Dual career policies for sub-elite athletes |  |  |
|  |  | Ensuring homogeneity between universities |  |  |
|  | Special access (reserved for actual or ex high sport performance practitioners) | Special access for High-level athletes | *"Dual career should be extended to all athletes/dancers/artists"* | |
|  |  | Special access for artists |  |  |
|  |  | Special access included in national legislation |  |  |
|  |  | Covered by mass media |  |  |
|  |  | Dedicated dual career programmes/webpages |  |  |
|  | Observatory at the university level (controlling and monitoring the application of the dual career progranne) | Web observatory | *“A sport observatory needs the creation of common guidelines among all Italian universities and a regular monitoring through common parameters. Even a national portal of dual career (with program, regulations, deadlines, etc.) would help a lot "* | |
|  |  | Hall of fame |  |  |
|  |  | Tutorship program |  |  |
|  |  | Implementing existant observatory |  |  |
|  |  | National database |  |  |
|  |  | Scientific research |  |  |
|  |  | Surveys |  |  |
|  |  | Collaboration among universities |  |  |
